# Intrathecal baclofen efficacy for managing motor function and spasticity severity in patients with cerebral palsy: a systematic review and meta-analysis

**DOI:** 10.1186/s12883-024-03647-7

**Published:** 2024-04-27

**Authors:** Mahdi Masrour, Amir Zare, Ana Presedo, Mohammad Hossein Nabian

**Affiliations:** 1 School of Medicine, Tehran University of Medical Sciences, Tehran, Iran; 2 Center for Orthopedic Trans-Disciplinary Applied Research, Tehran University of Medical Sciences, Tehran, Iran; 3 Department of Pediatric Orthopedics, Robert Debré University Hospital, Paris, France

**Keywords:** Intrathecal Baclofen (ITB), Cerebral palsy, Spasticity, Motor function, Ashworth scale, Gross Motor Function Measure (GMFM), Complications, Modified Ashworth Scale (MAS)

## Abstract

**Background:**

Spasticity can significantly affect a patient’s quality of life, caregiver satisfaction, and the financial burden on the healthcare system. Baclofen is one of only a few options for treating spasticity. The purpose of this study is to investigate the impact of intrathecal baclofen (ITB) therapy on severe40.23 spasticity and motor function in patients with cerebral palsy.

**Methods:**

We conducted a systematic review in PubMed, Scopus, Ovid, and the Cochrane Library in accordance with the PRISMA guidelines. We included studies based on eligibility criteria that included desired participants (cerebral palsy patients with spasticity), interventions (intrathecal baclofen), and outcomes (the Ashworth scales and the Gross Motor Function Measure [GMFM]). The within-group Cohen’s d standardized mean differences (SMD) were analyzed using the random effect model.

**Results:**

We screened 768 papers and included 19 in the severity of spasticity section and 6 in the motor function section. The pre-intervention average spasticity score (SD) was 3.2 (0.78), and the post-intervention average score (SD) was 1.9 (0.72), showing a 40.25% reduction. The SMD for spasticity reduction was − 1.7000 (95% CI [-2.1546; -1.2454], p-value < 0.0001), involving 343 patients with a weighted average age of 15.78 years and a weighted average baclofen dose of 289 µg/day. The SMD for the MAS and Ashworth Scale subgroups were − 1.7845 (95% CI [-2.8704; -0.6986]) and − 1.4837 (95% CI [-1.8585; -1.1088]), respectively. We found no relationship between the participants’ mean age, baclofen dose, measurement time, and the results. The pre-intervention average GMFM (SD) was 40.03 (26.01), and the post-intervention average score (SD) was 43.88 (26.18), showing a 9.62% increase. The SMD for motor function using GMFM was 0.1503 (95% CI [0.0784; 0.2223], p-value = 0.0030), involving 117 patients with a weighted average age of 13.63 and a weighted average baclofen dose of 203 µg/day. In 501 ITB implantations, 203 medical complications were reported, including six new-onset seizures (2.96% of medical complications), seven increased seizure frequency (3.45%), 33 infections (16.26%), eight meningitis (3.94%), and 16 cerebrospinal fluid leaks (7.88%). Delivery system complications, including 75 catheter and pump complications, were also reported.

**Conclusion:**

Despite the risk of complications, ITB has a significant impact on the reduction of spasticity. A small but statistically significant improvement in motor function was also noted in a group of patients.

**Supplementary Information:**

The online version contains supplementary material available at 10.1186/s12883-024-03647-7.

## Introduction

Spasticity is defined as hypertonia in conjunction with a velocity-dependent increase in tonic stretch reflexes to external force in joint movement [1]. For the sake of practicality, there are ongoing efforts to better define spasticity for clinicians and researchers. Notably, the Support Program for Assembly of a Database for Spasticity Measurement (SPASM) consortium defines spasticity as disordered sensory-motor control caused by an upper motor neuron lesion that manifests as intermittent or prolonged involuntary muscle contractions [2]. This manifestation is seen in a variety of neurological conditions, including cerebral palsy (CP), multiple sclerosis, spinal cord injuries, and brain injuries. Although not all patients are troubled by their spasticity, it can be associated with pain, sleep disturbances, and motor impairment. It can have a significant impact on the patient’s quality of life, as well as the satisfaction and ease of the caregiver, and place a significant burden on the healthcare system [3].

Some pharmacological treatments for spasticity, including baclofen, aim to increase inhibitory neurotransmitter levels in the spinal cord while decreasing excitatory neurotransmitter levels [4, 5]. Baclofen is an agonist for the neurotransmitter Gamma-Amino Butyric Acid (GABA-b) receptors on both pre- and post-synaptic neurons in the central and peripheral nervous systems. This drug inhibits reflex transmission at the level of the spinal cord, thereby alleviating spasticity [4]. While it has been established that oral administration of baclofen is effective in treating spasticity, there is growing recognition of the intrathecal administration of this medication as a potentially superior method. The difficulty with oral baclofen is that it is not well tolerated at higher doses due to the numerous side effects associated with it, such as severe sedation, confusion, muscle weakness, vertigo, nausea, and, in some cases, seizures and hallucinations. Hence, intrathecal delivery of baclofen could be a viable option for those with severe spasticity who find oral baclofen ineffective or intolerable; however, intrathecal administration also has a considerable risk of complications [5–7]. Intrathecal Baclofen (ITB) therapy involves the administration of medication through an implantable infusion system, which delivers the medication directly into the intrathecal fluid. This system comprises an implanted pump located in the abdominal region and a catheter that administers precise quantities of baclofen directly into the intrathecal space [8]. When administered intrathecally, baclofen bypasses the blood-brain barrier, delivering the medication at a higher concentration to the spinal and brain stem’s GABA-b receptors while avoiding the systemic side effects of oral baclofen [5, 9, 10]. ITB has demonstrated efficacy in reducing severe spasticity in patients for whom oral medications or botulinum toxin treatment are inadequate. Compared to the other approaches for managing non-responsive spasticity, namely orthopedic musculoskeletal surgery and selective posterior rhizotomy, ITB therapy has the added benefit of being reversible and providing continuous control over spasticity [11–13]. Given that CP is a collection of motor disorders, dystonia, rigidity, and other CP complications can also contribute to the observed resistance to movement, often described as tone [14]. It is worth noting that ITB is also used in the treatment of CP in patients with dystonia [15].

The primary aim of this research was to examine the impact of ITB therapy on the degree of spasticity and motor function in individuals diagnosed with cerebral palsy.

## Methods

### Search methods

On June 15, 2023, systematic searches in electronic databases, including PubMed, Scopus, Ovid, and the Cochran Library, were conducted using the search query provided in Supplementary Table 1. The bibliographies of studies and systematic and non-systematic reviews were reviewed to identify any relevant studies that had not been discovered through electronic database searches, and any missing suitable articles were added.

### Eligibility criteria

This review included studies in which at least half of the participants had spasticity due to a CP etiology and were treated with ITB. Studies involving continuous ITB administration via a spinal implant were considered eligible, regardless of the dosage or duration of treatment. All experimental studies, both prospective and retrospective, observational original studies, and systematic and non-systematic reviews were considered for inclusion. Studies without a standard deviation or confidence interval for the reported outcomes were excluded.

### Primary outcomes

1. The Ashworth-like Scales, including the Ashworth Scale and Modified Ashworth Scale (MAS), were used to assess the severity of spasticity [16]. Each scale has some properties and limitations [17–19].
2. Motor function was assessed using the Gross Motor Function Measure (GMFM). The GMFM is available in two editions: the original 88-item (GMFM-88) and the more modern 66-item (GMFM-66). Both variants were regarded as eligible [20].

### Secondary outcomes

Complications and side effects reported in the included studies and the frequency of their occurrence.

### Selection of studies

Following database searches and the removal of duplicate records, each title and abstract was reviewed and selected by two independent investigators (MM, AZ) in accordance with eligibility requirements. The full texts of the selected studies were then reviewed by investigators for inclusion. In the event of a disagreement, a third author was consulted.

### Data extraction

The following data was extracted independently by two investigators (MM and AZ) using the data extraction sheet developed by the team of authors:

- Authors, year of study, and study design.
- Demographic information about participants, including sample size, mean age and standard deviation, etiology and classification, and gender ratio.
- Comparators, baclofen dose, and measurement time.
- Study results, including average Ashworth scale and GMFM scores at baseline and after the intervention, along with their standard deviation or confidence interval (CI) and p-value for comparing before and after.
- Adverse events, complications, and side effects, as well as their frequency and severity, if mentioned.

### Assessment of risk of bias

To determine the level of evidence presented in this review, as well as the corresponding limitations and uncertainties, the Cochrane Risk of Bias tool, the Risk of Bias in Non-randomized Studies - of Interventions (ROBINS-I), was utilized to evaluate the studies included in the analysis [21]. Two independent authors (MM and AZ) assessed the risk of bias in each study. Based on the criteria provided by the Cochrane Handbook for Systematic Reviews of Interventions, the risks of bias in each reviewed study were classified as low, moderate, serious, or no information [22]. If disagreements could not be resolved through consensus, a third author (MHN) was consulted.

We additionally employed funnel plot analysis to assess reporting bias. A symmetrical funnel plot shows that there is no publication bias in this type of plot. Egger’s statistical analysis was also used to quantify publication bias [23].

### Statistical analysis

**Fig. 1:**
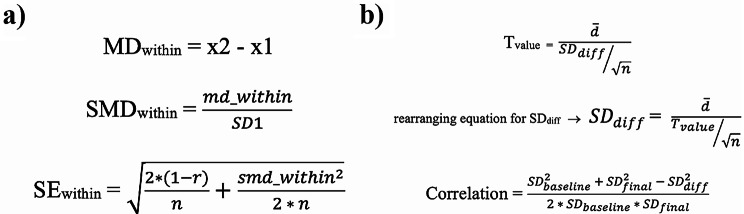
(**a**) within-group Cohen’s d; MD_within_ is the raw mean difference, SMD_within_ is the standardized mean difference, SE_within_ is the standard error, x1 and x2 are means from time 1 and time 2, SD1 is the standard deviation at time 1, r is the correlation between time 1 and time 2 and n is the sample size; (**b**) T_value_ is the paired t-test value, d is the average of differences of pairs, SD_diff_ is the standard deviation of the difference, n is the number of pairs

The statistical analysis was conducted using R version 4.2.2 (R Core Team [2021], Vienna, Austria). The random effect model was employed considering the variations in measurement techniques across studies and the anticipated heterogeneity in study outcomes. The within-group Cohen’s d statistic (Fig. 1) was utilized to compute the standardized mean difference (SMD) to compare the baseline and post-intervention measurements. Furthermore, the statistical significance cutoff for the study was set at p-values of 0.05. Higgins’ I-square test, which is based on Cochrane’s Q, was used to assess statistical heterogeneity. The thresholds for heterogeneity (I2) are set at 25%, 50%, and 75% for low, moderate, and high heterogeneity, respectively.

### Dealing with missing data

In the cases in which the correlation between the outcomes at baseline and post-intervention was not reported, we calculated it using the p-value and other measures reported. For this purpose, we first calculated the two-sided t-test values from p-values. We then calculated the standard deviation of the difference by rearranging the paired t-test formula. Using the standard deviation of the difference, along with the standard deviations of the baseline and post-intervention scores, the correlation of the pairs was calculated according to the Cochran handbook method (Fig. 1).

When a study reported the median and interquartile range, the mean and standard deviation were determined using statistical methods developed by Luo et al. [24].

Studies that did not provide standard deviations, confidence intervals, or any associated values for pre- or post-intervention scores were excluded.

## Results

### Search results

**Fig. 2:**
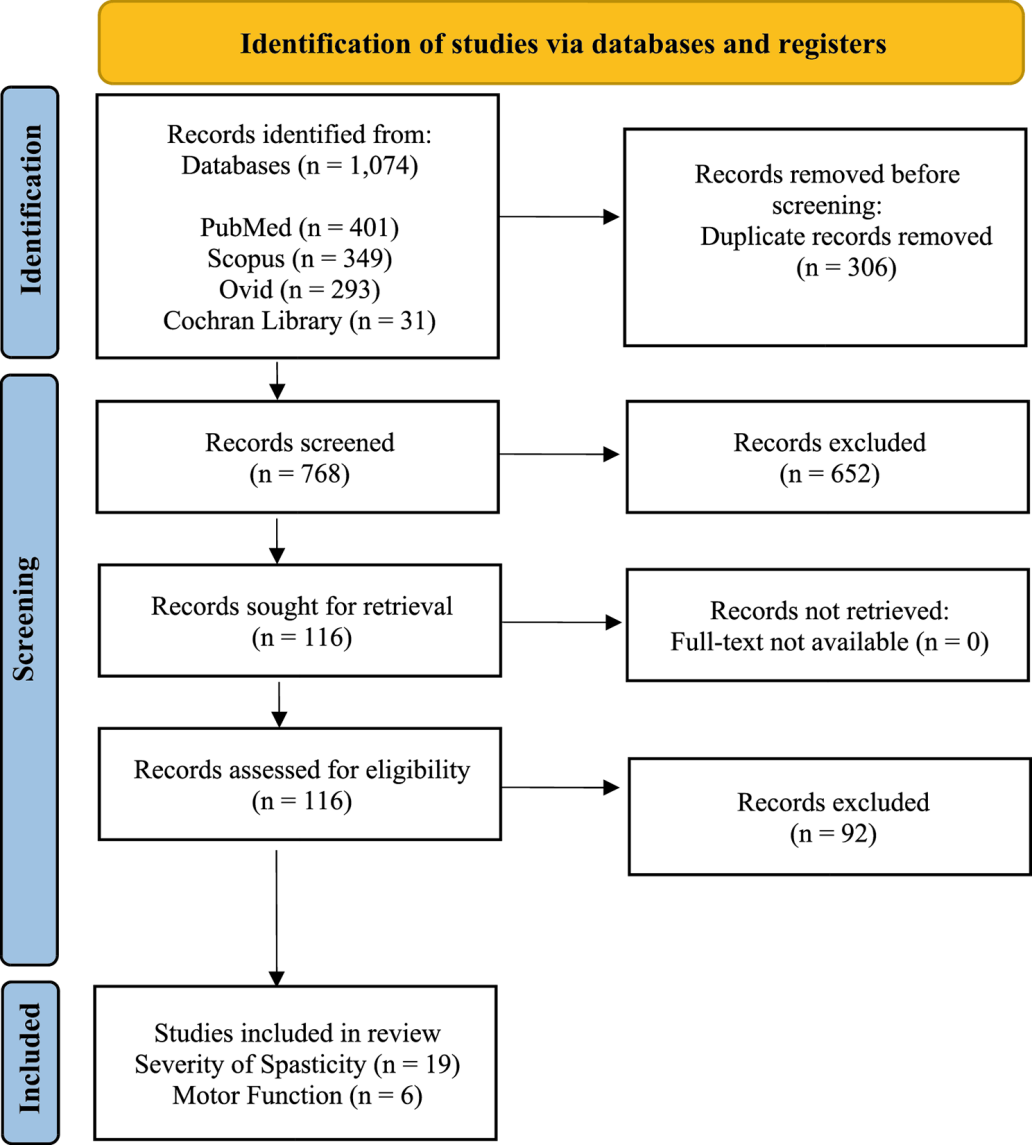
PRISMA Flowchart

The initial database search yielded 1,074 results. After removing duplicates, we screened the remaining 768 abstracts and excluded 652. There were 116 articles left for full-text review. Nineteen of these met the inclusion criteria for the severity of spasticity, and six met the criteria for the motor function sections. The PRISMA flowchart explains the details of study inclusion and exclusion (Fig. 2).

### Basic characteristics

The details and characteristics of the studies and subjects included in the meta-analysis are summarized in Table 1. The papers included in the spasticity severity section have been published between 1997 and 2020. 343 patients from these papers were included in the meta-analysis, with a weighted average age of 15.78 years and a 61.51% male ratio. Two patients from these studies had diagnoses other than CP, but we were unable to separate them from the CP patients. The papers included in the section on motor function were released between 2005 and 2015. The meta-analysis included 117 patients from these studies, all with CP diagnoses, with a weighted average age of 13.63 and a male-to-female ratio of 55.56%. The majority of the participants in the included studies were non-ambulatory (Table 1).

**Table 1:** Characteristics of included studies

Severity of Spasticity																
Study	Total Included Cases	Mean Age (SD)	GMFCS level^#^ or ambulatory status								male %	% of CP patients *	Average measurement time	Measurement Scale	Average Final dose (µg/day)	Dose Details
			I	II		III		IV		V						
Barney et al., 2020 [66]	28	9.9 (3.08)		3.1%		6.3%		12.5%		78.1%	53%	100%	5.61	Modified Ashworth Scale		
Simsek et al., 2018 [67]	8	10.63 (2.33)	All non-ambulant								88%	100%	12.88	Modified Ashworth Scale	81.38	50–140 µg/day
Yoon et al., 2017 [68]	8	36.28	57.9% non-ambulant								74%	100%	12.00	Modified Ashworth Scale		Bolus: 50 µg on the first day, 75 µg on the second day, 100 µg on the third day (if the second-day dose was ineffective)
Dekopov et al., 2015 [69]	8	25.88 (9.06)									60%	100%	14.00	Ashworth Scale	214.00	50–75 µg/day (the rate was daily increased by 15—20% of the total dose until a clinical effect was achieved)
Walter et al., 2014 [70]	15	12 [4]							23.1%	76.9%	33%	87%	42.00	Modified Ashworth Scale	439.00	25–820 µg/day
Margetis et al., 2014 [71]	8	30.38 (9.05)									75%	100%	12.00	Modified Ashworth Scale	156.30	60–380 µg/24 h
Gray et al., 2012 [72]	37	10.16 (3.25)						48.6%		51.4%	51%	100%	18.00	Modified Ashworth Scale	250.00	50–650 µg/24 h (9 months), 35–1000 µg/24 h (18 months)
Ramstad et al., 2010 [73]	28	8.58				5.7%		37.1%		57.2%	71%	100%	18.00	Modified Ashworth Scale	157.00	range 86–576 µg ⁄ day
Tassëel Ponche et al., 2010 [26]	25	29.6 (12.66)	All wheelchair-dependent: Third-party dependent 76%, Independent 24%								56%	100%		Modified Ashworth Scale	292.00	25–1015 µg per day (292 ± 106.4 µg per day)
Brochard et al., 2009 [74]	7	15 (5.4)		42.9%		57.1%					29%	100%	16.00	Ashworth Scale	121.80	range 75–250 µg ⁄ day
Motta et al., 2008 [75]	20	11.4 (3.57)		10%		40%		35%		15%	75%	100%	12.00	Ashworth Scale		
Awaad et al., 2003 [76]	21	13.69 (7.43)									69%	100%	18.00	Ashworth Scale	563.23	50-µg bolus, 75–1196 µg/day
Murphy et al., 2002 [77]	23	8.8 (3.9)	57.1% non- ambulant								74%	100%	6.00	Ashworth Scale		
Meythaler et al., 2001 [78]	13	25.46 (10.15)									77%	100%	12.00	Ashworth Scale	263.00	160–470 µg/d
Gilmartin et al., 2000 [79]	40	10.30									57%	100%	12.00	Ashworth Scale	265.20	50-µg bolus, 25 µg/day to 1350 µg/day
Avellino et al., 2000 [80]	4	28.3 (5.3)									75%	100%	12.00	Ashworth Scale	460.80	460.8 ± 345.6 µg/day
Wiens et al., 1998 [25]	17	10.20									59%	100%	3.00	Ashworth Scale		
Armstrong et al., 1997 [81]	10	10.42 (4.45)											12.00	Ashworth Scale	466.18	
Creedon et al., 1997 [82]	23	35.6 (11.7)										100%	19.60	Ashworth Scale	305.00	
Motor Function section																
Dekopov et al., 2015 [69]	7	25.88 (9.06)									60%	100%	14.00	GMFM-88	214.00	50–75 µg/day (the rate was daily increased by 15—20% of the total dose until a clinical effect was achieved)
Gray et al., 2014 [83]	23	10.83 (3.5)				13.0%		65.3%		21.7%	70%	100%	9.00	GMFM-88	158.00	50 to 530 µg/day
Motta et al., 2011 [84]	37	13.58 [7]		24.3%		35.2%		18.9%		21.6%	49%	100%	12.00	GMFM-88		
Hoving et al., 2009 [85]	12	13.7 (2.9)				5.9%		11.8%		82.3%	47%	100%	12.00	GMFM-66	232.76	61–233 µg/day
Bleyenheuft et al., 2007 [86]	7	18.4 [7]	71.4% dependent on a wheelchair most of the day (spasticity preventing ambulation), 28.6% non-ambulant								29%	100%	14.29	GMFM-66	110.57	external 45–150 µg/24 h, internal 66–160 µg/24 h
Krach et al., 2005 [87]	31	11.90	Ambulatory without devices: 3.2%, ambulatory with orthoses or devices: 22.6%, mobility by crawling on hands and knees or independent wheelchair: 16.1%, non-ambulatory however having other purposeful motor activity: 35.5%, minimal or no purposeful motor activity: 22.6%								61%	100%	12.00	GMFM-88	242.00	73–242 µg/day

* Although we attempted to exclude non-CP patients from some studies, this was not always possible, so we have listed the proportion of CP patients from each study who were included in our meta-analyses

# GMFCS data are reported for the total population, not just CP patients. I (ambulant without assistance), II (ambulant without assistive devices, limitations outside the home), III (ambulant with assistive devices, wheelchair required outside home), IV (non-ambulatory, self-mobile in a wheelchair with limitations), V (non-ambulatory, self-mobility very limited)

### Risk of bias

**Fig. 3:**
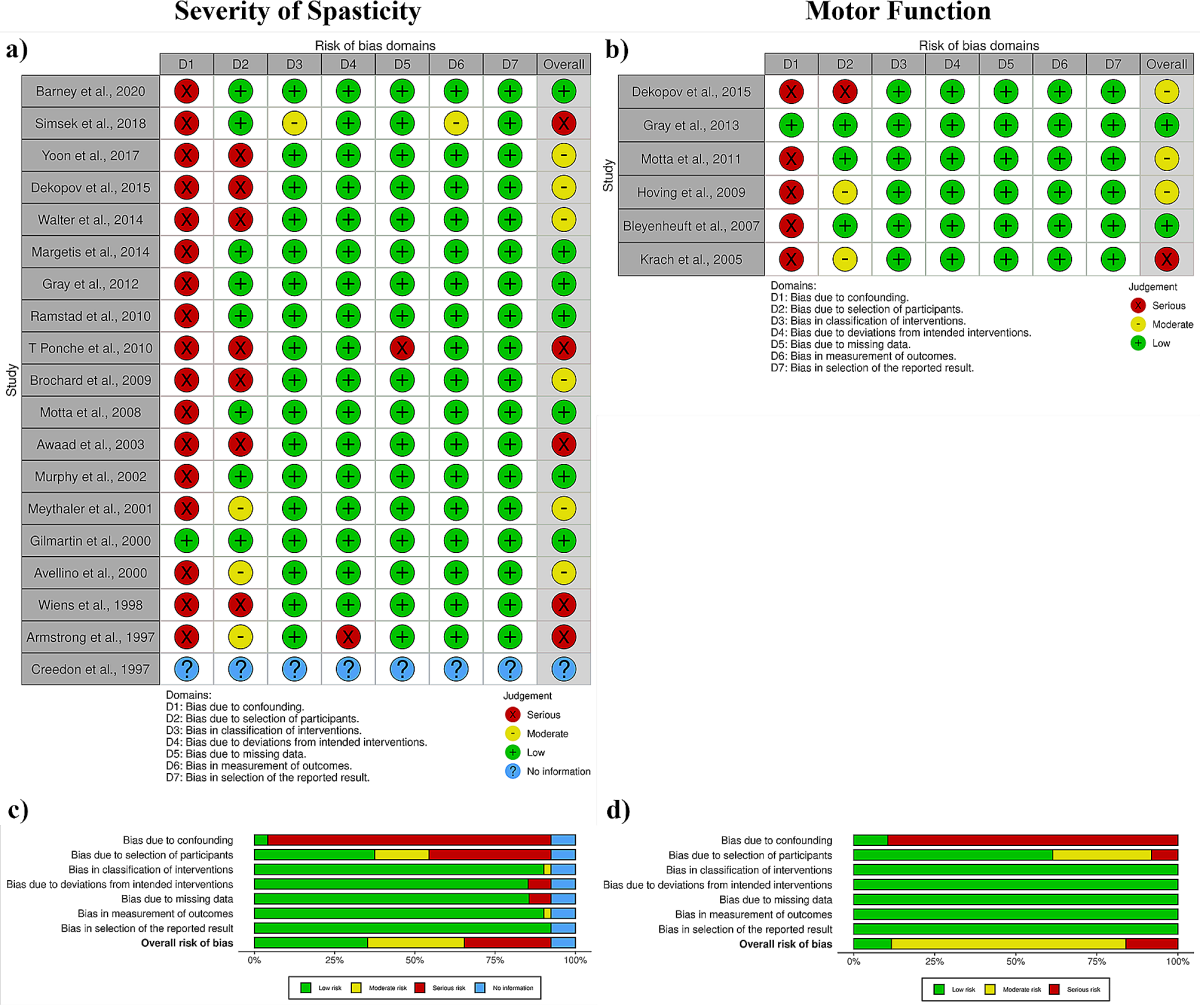
(**a**) and (**c**) risk of bias scores and graph for the severity of spasticity; (**b**) and (**d**) risk of bias scores and graph for motor function

Independent investigators assessed the quality of the included studies using the ROBINS-I tool [21]. Any disagreements in the quality assessment were resolved by a third investigator. Figure 3 depicts the results of the quality assessment of the included studies in the form of a Cochrane risk of bias graph, as well as the risk of bias scores for each section. The most biased aspects of the included studies were confounding and participant selection, which may have influenced the meta-analysis results. The overall risk of bias score depicted in Fig. 3 is calculated using the Cochrane tool.

The contour-enhanced funnel plots for the motor function and severity of spasticity parts of this systematic review are shown in Supplementary Fig. 1. The asymmetrical funnel plots indicate the presence of publication bias. Furthermore, Egger’s tests revealed significant evidence of publication bias among the included studies (severity of spasticity p-value = 0.000596 and motor function p-value = 0.0336).

### Effects of interventions

#### Spasticity severity

In our meta-analysis, eight of the 19 studies reported MAS scores, while the remaining studies reported Ashworth Scale scores. Overall, ITB therapy was effective in reducing spasticity, with both subgroups showing decreased levels of post-intervention spasticity and a statistically significant decrease in scale scores following ITB pump implantation. The pre-intervention average spasticity score (SD) was 3.2 (0.78), and the post-intervention average score (SD) was 1.91 (0.72), showing a 40.25% reduction. ITB pump implantation was linked to statistically lower levels of spasticity, with a pooled SMD of -1.7000 (95% CI [-2.1546; -1.2454], p-value < 0.0001) for all studies combined. Statistical heterogeneity between studies was also significant (I2 = 72.1%, p-value < 0.0001). The weighted average final baclofen dose for the 14 studies that have reported it was 289 µg/day.

**Fig. 4:**
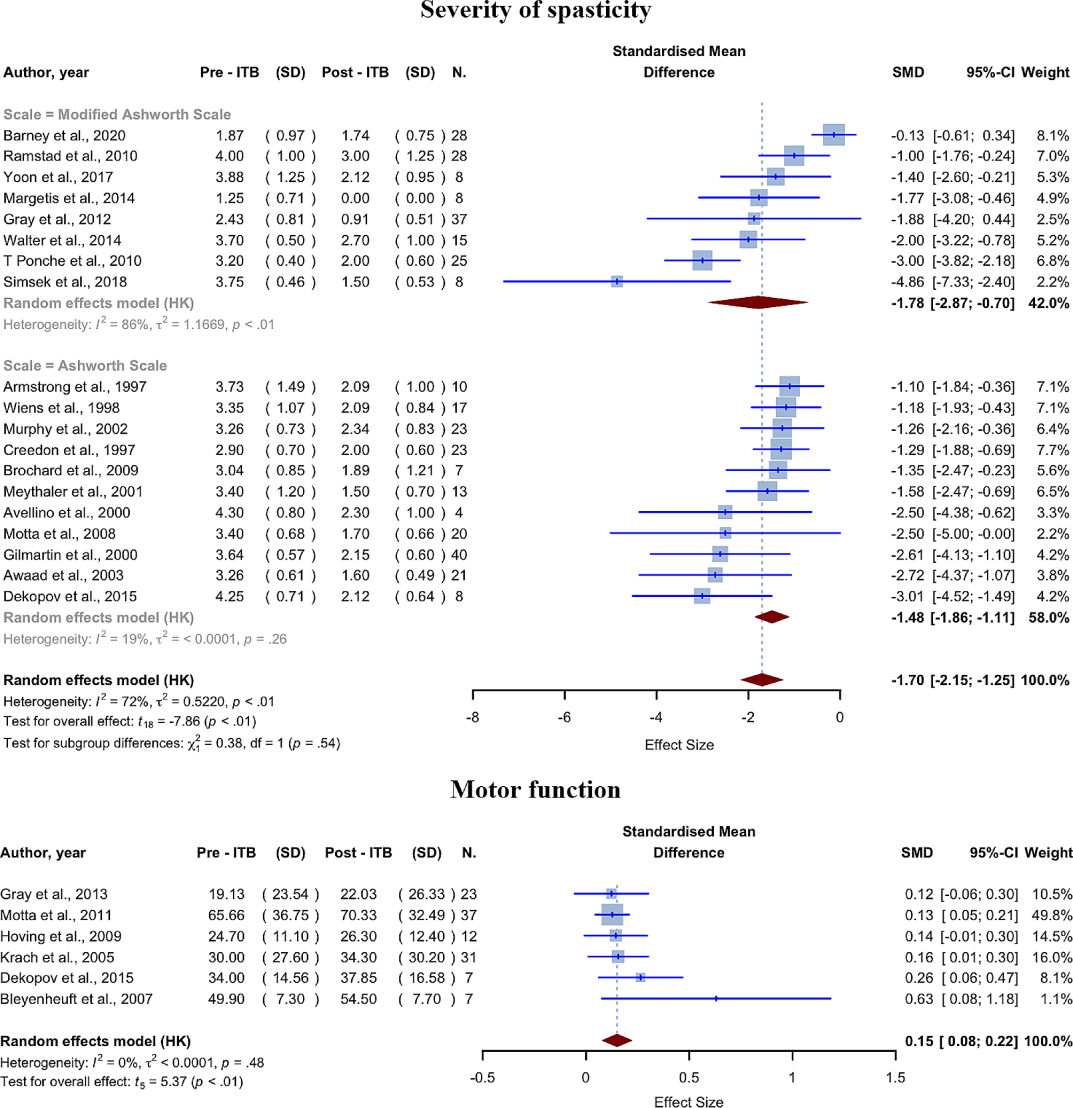
Forest plot for severity of spasticity and motor function sections; Pre-ITB (SD) refers to the mean score and standard deviation prior to ITB implantation, whereas post-ITB (SD) refers to mean scores after implantation. N. represents the number of participants in the research

The pre-intervention average score (SD) for the MAS subgroup (eight studies and 157 participants) was 2.93 (0.78), and the post-intervention average MAS score (SD) was 1.82 (0.74), showing a 37.95% reduction. The pre-intervention average score (SD) for the Ashworth Scale subgroup (eleven studies and 186 participants) was 3.41 (0.79), and the post-intervention average Ashworth Scale (SD) was 1.98 (0.71), showing a 41.90% reduction. The SMD for the MAS subgroup was − 1.7845 (95% CI [-2.8704; -0.6986], I2 = 85.9%), and the SMD for the Ashworth Scale subgroup was − 1.4837 (95% CI [-1.8585; -1.1088], I2 = 19.2%) (Fig. 4). The test for subgroup differences revealed no significant differences between the MAS and Ashworth Scale groups (p-value = 0.5385). It is important to highlight that, generally, the implantation of an ITB pump was primarily reserved for patients exhibiting Ashworth scores of 4 or 5. As a result, the range of pre-ITB scores was relatively narrow. Therefore, the low standard deviation influences the magnitude of the effect and the weight of the studies in meta-analysis.

A multiple meta-regression was used to identify potential influencing factors and sources of heterogeneity between studies. The meta-regression analysis revealed no statistically significant relationship between the participants’ mean age, baclofen dosage, time of measurement, and effect size (Table 2).

**Table 2:** Multiple meta-regression results

Multiple meta-regression for severity of spasticity section									
Moderator		No. of comparisons	No. of subjects		Meta-regression Slope		95% CI		*p*-value
Mean age		19	343		0.0006		− 0.0556	0.0568	0.9801
Measurement time		18	318		0.0011		− 0.0767	0.0790	0.9749
Baclofen dose		14	247		− 0.0001		− 0.0045	0.0044	0.9736
**Multiple meta-regression for motor function section**									
Mean age	6			117		-0.0095	-0.0469	0.0278	0.1906
Measurement time	6			117		0.0863	-0.0548	0.2274	0.0815
Baclofen dose	5			80		-0.0027	-0.0069	0.0016	0.0799

**Fig. 5:**
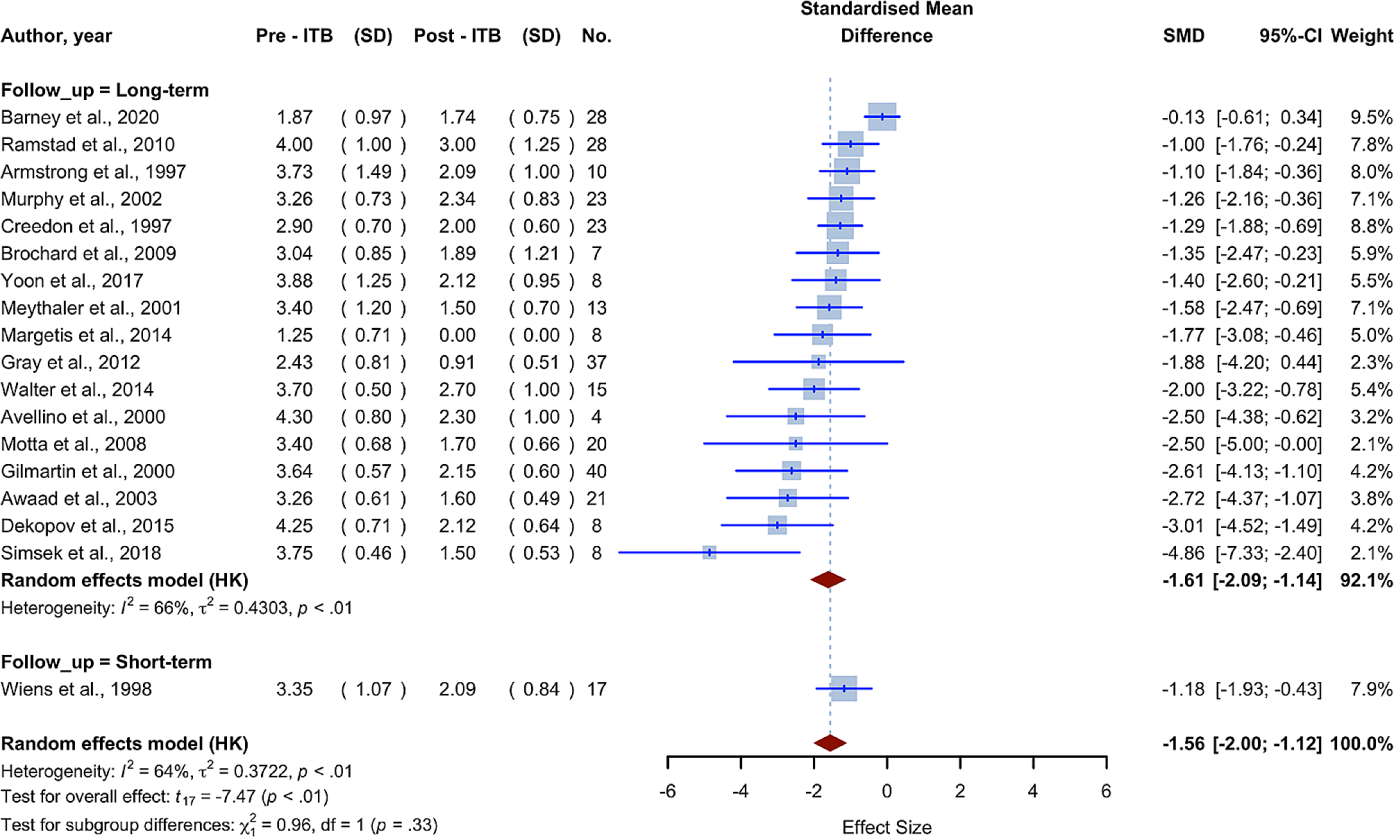
Forest plot for severity of spasticity studies subgrouped into short-term and long-term follow-up studies; Pre-ITB (SD) refers to the mean score and standard deviation prior to ITB implantation, whereas post-ITB (SD) refers to mean scores after implantation. No. Represents the number of participants in the research

Generally speaking, given the dose adjustment procedure, the baclofen dose can vary significantly in the first few months following ITB implantation. Of the studies we have included in this section, Wiens et al. [25] reported a follow-up period of three months, while Tassëel Ponche et al. [26] did not report on the follow-up period or the time after ITB implantations that data were collected about patients. In order to investigate the significance of the dose adjustment period, we divided the studies into short-term and long-term subgroups after excluding the Tassëel Ponche et al. study. The short-term subgroup involved only the Wiens et al. study, which had a follow-up time of less than six months. The SMD for the long-term subgroup was − 1.6124 (95% CI [-2.0872; -1.1376], I2 = 65.9%), and the SMD for the short-term subgroup was − 1.1776 (95% CI [-1.9288; -0.4264]) (Fig. 5). The test for subgroup differences found no statistically significant differences between the short-term and long-term subgroups (p-value = 0.3273), indicating that follow-up time had little impact on our study’s findings. It is worth noting that the combined SMD of long-term studies and all studies combined is very similar (-1.6124 vs. 1.7000).

#### Motor function

GMFM scores were reported in six studies. The findings from all six studies indicated that participants exhibited elevated levels of motor function following the implementation of ITB treatment. When the pre- and post-ITB treatments were compared, it was shown that there was a small but statistically significant mean difference in motor function scores. The pre-intervention average GMFM (SD) was 40.03 (26.01), and the post-intervention average GMFM score (SD) was 43.88 (26.18), showing a 9.62% increase. ITB pump implantation was linked to statistically higher levels of GMFM, with an SMD of 0.1503 (95% CI [0.0784; 0.2223], p-value = 0.0030, Fig. 4). There was no statistically significant heterogeneity between studies (I2 = 0.0%, p-value = 0.4793). The weighted average final baclofen dose for the five studies that have reported it was 203 µg/day.

To identify potential influencing factors and sources of heterogeneity between studies, a multiple meta-regression was used. The meta-regression analysis revealed that there was no statistically significant relationship between the participants’ mean age, baclofen dosage, measurement time, and effect size (Table 2).

#### Safety and complications

A total of 501 participants (some did not have CP) were evaluated for complications in the included studies from both the spasticity severity and motor function sections. Among the adverse events reported in the studies, 75 were catheter or pump complications, and 203 were medical complications. Seizure-related complications were of significant concern among the reported adverse events. A total of 6 instances of new-onset seizures (2.96% of medical complications) were reported among the entire patient population, resulting in an event incidence per-person rate of 0.012 (6/501). Additionally, there were seven instances of increased seizure frequency (3.45% of medical complications) reported, resulting in an event incidence per person rate of 0.014 (7/501). Infection, primarily originating from wounds, as well as meningitis, both of which are serious conditions for patients with CP, were observed in 33 (16.26% of medical complications) and 8 (3.94% of medical complications) instances, respectively, with per-person incidences of 0.066 (33/501) and 0.016 (8/501). Cerebrospinal fluid leaks are another serious complication of ITB implementation, which were reported in 16 cases (7.88% of medical complications), accounting for a per-person incidence of 0.032 (16/501). In our analysis, catheter and pump complications, specifically dislocations, malfunctions, and disconnections, were identified as the most common complications. These complications were observed in 75 events, resulting in a 0.15 (75/501) per-person incidence rate. Supplementary Table 2 summarizes the complications observed in the included studies.

#### Interpretation of the results

Table 3 summarizes our findings and interpretations of the meta-analyses that were conducted. The SMD is a method of calculating the amount of an intervention’s effect or the difference between two groups. Our SMD interpretation is based on Cohen’s criteria, where 0.2 is a small difference, 0.5 is a moderate difference, and 0.8 is a large difference [27]. If the CI contains a zero, it indicates that there was no significant difference at the selected threshold of significance (p-value < 0.05). None of the SMD CIs in our meta-analyses included 0.

**Table 3:** Summary of findings

	Number of studies	Number of Participants	Mean Age	Weighted Average Final Dose (µg/daily)	Pre-intervention weighted average(SD)	Post-intervention weighted average(SD)	Average Difference %	SMD	SMD Interpretation
**Spasticity severity, Ashworth Scale**	11	186	16.00	333	3.41 (0.79)	1.98 (0.71)	-41.90%	-1.48	Very large difference
**Spasticity severity, Modified Ashworth Scale**	8	157	15.45	243	2.93 (0.78)	1.82 (0.74)	-37.95%	-1.78	Very large difference
**Total**	**19**	**343**	**15.78**	**289**	**3.2 (0.78)**	**1.91 (0.72)**	**-40.25%**	**-1.70**	Very large difference
**Motor function, GMFM score**	**6**	**117**	**13.63**	**203**	**40.03 (26.01)**	**43.88 (26.18)**	**9.62%**	**0.15**	Small difference

According to the MAS and Ashworth Scale, ITB therapy significantly reduced the severity of spasticity in CP patients. The average difference percentage was − 41.90% for the Ashworth Scale and − 37.95% for the MAS, indicating a large improvement in spasticity after ITB therapy. The SMD was − 1.48 for the Ashworth Scale and − 1.78 for the MAS, indicating a “large difference” between pre- and post-intervention. ITB therapy also improved motor function in patients with CP, as measured by the GMFM score. The average difference percentage was 9.62%, indicating a small but measurable improvement in motor function after ITB therapy. The SMD was 0.15, showing a “small statistically significant difference” between pre- and post-intervention.

## Discussion

### Summary of main results

#### Spasticity severity

The Ashworth score improved after pump implantation in all studies included in this review, but the degree of improvement varied. The weighted mean reduction in spasticity after ITB implantation was a 1.28 score on the Ashworth-like scales. The pre-treatment weighted average of the Ashworth-like score was 3.19, implying that ITB reduced the average spasticity of participants by 40.25%. The calculated − 1.70 overall SMD showed a statistically significant difference between before and after treatment scores, indicating that ITB is effective in reducing the extent and severity of spasticity. This decrease was observed over a weighted average measurement period of 15.85 months. Based on the exact scaling tool used, we divided the studies into MAS and Ashworth scale subgroups. The effect sizes were close for these subgroups, indicating that the degree of improvement on both scales was comparable and that there were no statistically significant differences between subgroups. The MAS subgroup had significantly higher heterogeneity than the Ashworth scale subgroup, possibly indicating bias in this type of measurement. It is worth noting that certain medical centers will only adopt ITB in patients with an Ashworth score of 2 or above, while others will begin with a score of 3 or above. The lower the initial score for ITB implementation, the greater the floor effect on the intervention outcome. So the statistical heterogeneity seen in the meta-analysis might be attributed to the inclusion criteria (degree of spasticity) for admission to the therapy and the resultant floor effect. The meta-regression showed that the mean age, time of measurement, or baclofen dosage did not have a statistically significant effect on how well the intrathecal pump reduced spasticity.

#### Motor function

It is crucial to highlight that the notion of “functional status” differs from the term “function” as employed in the GMFM or other comparable instruments utilized in our study. These instruments evaluate certain areas of function, such as mobility in the case of GMFM, but do not offer a complete picture of a patient’s total functional state, as defined by the FDA [28]. Readers should be aware that whenever we used the terms “motor function” or “function” in this study, we were referring only to the physiological aspects of mobility and motor movements.

The challenge with attempting to improve motor performance in CP patients is that many have very little voluntary motor control; therefore, reducing spastic hypertonia may not have a significant impact on the motor score. Nevertheless, in all studies included in this review, the GMFM score increased after pump implantation, but the magnitude of the improvement varied. ITB improved GMFM after 11.67 months (weighted mean) of measurement time, but the improvement was not as significant as the reduction in spasticity. The weighted mean GMFM score improved by 3.86 points following ITB implantation. Before ITB, the weighted average of the GMFM scores was 40.03. This improvement in mean GMFM represented an improvement of 9.62%. ITB was effective in improving motor function but had a limited range of capability, as shown by the calculated 0.15 SMD, which also showed a statistically significant but small difference between pre- and post-treatment results. The studies used two slightly different scales, the GMFM-66 and GMFM-88. As only two studies were present in the GMFM-66 group, we did not conduct a subgroup analysis for these scales, but the level of improvement in both scales was comparable, and there were no statistically significant differences between them. Furthermore, because there were fewer studies in the GMFM-66 subgroup, this group displayed more heterogeneity. Additionally, according to the meta-regression, neither the mean age, the timing of the measurement, nor the dosage of baclofen had a statistically significant effect on how well the intrathecal pump reduced spasticity.

Given that the majority of the patients involved in the studies were non-ambulant, it is reasonable to infer that a 9.62% improvement in GMFM with an SMD of 0.15 is a significant improvement in the patient’s mobility.

Although our study focused on CP patients with spasticity, since it can be difficult to differentiate between spasticity and dystonia at times, it is essential to compare our findings with the published study on the efficacy of ITB for dystonia in CP patients. Our findings contradict the previously cited study, which claimed that ITB may improve dystonia and pain, as well as the achievement of individualized goals and quality of life, but may not improve motor function. There was no statistically significant improvement in motor function throughout the studies evaluated, as demonstrated by an SMD of 0.13; 95% CI − 0.33 to 0.59 (95% CI [− 0.33; 0.59], p-value = 0.57) [29]. This might imply that ITB is a better choice for spastic CP patients compared to dystonic CP patients.

### Safety and complications

ITB carries with it a high likelihood of adverse effects that require attention and monitoring [30–32]. We classified the reported ITB complications in the included studies based on their severity and importance into minor and major complications categories for each body system. Table 4 depicts and summarizes the classification results.

**Table 4:** ITB Complications

System	Major Side Effects	Minor Side Effects	References
Immune System	Infections, Meningitis		[26, 68, 70, 73, 76, 77, 79–81, 84, 86]
Nervous system	Seizures, Cerebrospinal fluid leaks	Drowsiness, Sleepiness, Headache, Increased oral secretions, Lethargy	[25, 26, 68, 72, 76–80, 84–86]
Cognitive system	Hallucinations, Agitation, Depression, Irritability, Other psychiatric side effects		[25, 73, 80, 81]
Cardiovascular system	Hypotension, Bradycardia, Fainting	Edema of ankles, feet, or upper libs, Hypothermia	[26, 67, 72, 81, 85]
Respiratory system	Respiratory depression, Apnea		[81]
Gastrointestinal system	Nausea/vomiting	Constipation, Reflux	[26, 72, 76–78]
Urinary system	Urinary retention	Dysuria, Cystitis	[26, 72, 76, 85]
Skin	Cutaneous hematoma, Seroma	Skin reactions, Pressure sore, Wound dehiscence, Pruritus	[25, 26, 68–70, 72, 74, 76, 77, 79, 85]
Muscular system	Progression of scoliosis	Decreased balance, Flaccidity, Jumpy legs	[25, 68, 72, 78, 81]

Drug-related side effects, including baclofen withdrawal or overdose symptoms, are among the highly preventable complications. If the pump stops working, runs out of medication, or malfunctions, baclofen withdrawal may happen. Severe withdrawal symptoms, including high body temperature, mental confusion, rigid muscles, convulsions, and even death, are possible. It is crucial to strictly adhere to the manufacturer instructions, keep the scheduled refill appointments, and address any withdrawal symptoms as soon as possible [32–34]. On the other hand, if the pump administers too much medication, which can happen due to software errors, human error, or device failures, an overdose can occur. Overdose symptoms include fatigue, lightheadedness, nausea, vomiting, low blood pressure, headaches, seizures, and muscle weakness. It is also crucial to monitor the patient for overdose warning signs and even de-implant the pump if necessary [32, 34–38]. Although overdoses due to over-infusions have been reported in the literature on ITB, Medtronic, the manufacturer of SynchroMed II pumps used for intrathecal drug administration, has reported 46 cases out of 10,053 total patients, which is extremely uncommon. Nonetheless, this information is not limited to ITB or spasticity and includes other applications of SynchroMed II pumps [32, 39]. Complications caused by the device may disrupt medicine administration and result in withdrawal or overdose symptoms. These include catheter dislodgement, twisting, cracking, or leaking; failure of the pump or battery depletion; and skin erosion above the pump. These conditions may necessitate surgical revision or device replacement, so it is critical that the pump and its logs be reviewed on a regular basis to ensure that there have been no motor stalls and that the device has been functioning properly since the patient’s previous visit [32, 40–42].

At any time in the course of ITB admiration, infection may arise at the implantation site or along the catheter that links the pump to the spinal canal. Symptoms of infection include redness, swelling, discomfort, fever, and pus [32, 43–47]. Pump replacements are also prone to infection. When a replacement pump is implanted where the old pump left scar tissue, infection typically arises due to the poor circulation of scar tissue [48, 49]. In rare situations, ITB may also cause meningitis or abscess development. It is critical to keep the site clean and dry, to avoid bathing in contaminated water, and to treat any infection as soon as possible [32, 38, 50].

Collectively, ITB is not without risks and must be carefully managed and followed up on. There are certain methods for troubleshooting and avoiding ITB complications. Patients and caregivers should be educated about the benefits and risks of ITB therapy and be prepared to deal with any adverse events or complications that may arise. Patients with suboptimal ITB therapy effects should be evaluated immediately through an extensive medical history, physical examination, and para-clinical testing [51].

### Applicability for practice

ITB therapy involves the delivery of baclofen into the spinal fluid through a pump implanted under the skin. This allows for targeted delivery of the medication to the affected area, resulting in better spasticity control with fewer side effects compared to oral medication [52, 53]. Our meta-analysis findings suggest that ITB may be a safe and effective treatment for spasticity caused by CP. The available clinical evidence for using ITB to treat spasticity is quite adequate in quantity, with the majority of studies demonstrating significant improvements in spasticity and motor function. However, ITB therapy is not without risks and complications. Infection at the pump site and meningitis, catheter and pump malfunction or dislodgement, seizures onset or increase, and cerebrospinal fluid leakage are the common side effects. The pump and catheter systems must be monitored and maintained regularly to ensure proper operation. It should be noted pump and catheter-related complications were largely preventable with improved surgical abilities and careful pump and catheter placement techniques, as shown in other studies [40, 42]. To minimize risks and ensure optimal outcomes, however, careful patient selection and regular monitoring are required [54].

As complications will often occur following ITB implantation, with the catheter being the most common source, to maximize treatment success and allow early detection of system-related problems, ITB system care should be managed by a specialized and experienced team of specialists, including surgeons, physicians, nurses, therapists, etc. Studies have found that intrathecal drug delivery catheter complication incidence varies greatly between medical centers, implying that surgical implantation procedures may account for these disparities [55, 56]. Standardized documentation of ITB treatment should be kept and made available to all clinicians involved in the administration of ITB therapy, especially when dealing with complications. Patient and family education for the detection of adverse effects should also be addressed since this is crucial in avoiding or minimizing serious side effects [55, 57].

As seen in studies, the dosage of baclofen will be increased gradually after implantation. These gradual dosage increases are necessary to minimize potential negative effects while carefully managing the patient’s spasticity and providing motor control [58]. The patient population that required the highest starting doses also required the highest total dosage increases during treatment. Future guidelines for pump implantation will need to consider this need for the titration aspect of the therapy and may need to add an adjustment factor based on the first dose required to produce a therapeutic response in the long run. To best determine starting doses and their relationship to the final titrated dose for patients, more research is also required [32, 34, 38].

Although multiple studies have demonstrated that ITB works efficiently in treating severe spasticity, fewer studies have demonstrated that this therapy provides a greater benefit in motor function results when compared to more traditional therapies, and this is what further studies on this method of therapy must address [59, 60]. While there are some disagreements about the cost-benefit of ITB in the literature, it remains a significant treatment option for some patients with severe spasticity who have not responded to other treatments [61, 62].

### Limitations of the study

Despite extensive study and evaluation in clinical trials, the majority of studies have a low level of evidence, which makes it difficult to draw definite conclusions on the effects of continuous ITB in CP patients. The lack of double-blinded placebo-controlled studies on ITB will remain problematic. Furthermore, the current level of evidence for ITB reflects the limitations of research without a control group and necessitates additional efforts to conduct controlled studies. There is also a lack of consensus on the outcome metrics and scales used to evaluate the intervention results [17–19], as well as small and diverse patient populations and a scarcity of data for studies on chronic ITB use. Despite these constraints, treatment has been shown to effectively reduce spasticity and improve the motor function of CP patients, though individual results may vary.

It is also important to mention that, due to reporting limitations in the included studies, our study was not able to separate ambulant and non-ambulant patients for analysis of motor function. Future studies have to subcategorize patients based on the Gross Motor Function Classification System (GMFCS) and report data on motor function measures, such as GMFM, for these groups separately, as the primary goal of treating patients with CP is to improve function in the GMFCS II and III groups [63].

### Further research

Patients with differences in body size, metabolism, and severity of diseased or ambulatory stages may react differently to ITB; hence, it is essential to determine potential influencing factors and appropriate dosages for them. Identifying subgroups of patients with CP who are most likely to benefit from ITB therapy can also help clinicians make informed decisions about who should receive therapy. Long-term outcomes of ITB therapy in patients with CP are also needed, as short-term studies have shown improvements in spasticity and motor function, but it is unclear if these benefits are sustained over time. Long-term studies can also help determine if ITB therapy can prevent or delay secondary complications associated with spasticity, such as joint contractures or scoliosis. Although there are studies on scoliosis following ITB, the issue they address is whether or not ITB accelerates scoliosis, and ITB is not contraindicated in patients with scoliosis. Additionally, alternative delivery methods for baclofen, such as intranasal or transdermal administration, may provide additional treatment options for patients with CP, as attempts to develop an intravenous preparation of baclofen are underway [64, 65].

## Conclusion

Our meta-analysis demonstrated that ITB can be an effective treatment for severe spasticity, but it has a significant side effect profile. The lack of double-blind placebo-controlled studies, non-randomized research limitations, and consensus on outcome metrics and scales make it difficult to draw firm conclusions. Nonetheless, ITB can improve patients’ mobility and lead to spasticity control. Future studies should focus on implementing randomization and control to the extent that the nature of the problem permits. Further research is also needed to determine potential influencing factors, identify subgroups of patients who may benefit more, and determine long-term outcomes. Additionally, alternative delivery methods for baclofen may provide additional treatment options for CP patients. Finally, comprehensive guidelines are required to determine the best protocol for initiating and managing patients with severe spasticity caused by CP.

### Electronic supplementary material

Below is the link to the electronic supplementary material.

Supplementary Material 1

Supplementary Material 2

## Data Availability

The dataset(s) supporting the conclusions of this article is(are) included within the article (and its additional file(s)).

## Declarations

### Registration details

This study is a systematic review with no registration number or other identifying information.
