# Supplementary material for: Intrathecal baclofen efficacy for managing motor function and spasticity severity in patients with cerebral palsy: a systematic review and meta-analysis
Source: BMC Neurol. 2024 Apr 27;24:143. doi: 10.1186/s12883-024-03647-7 (PMC11055284; doi:10.1186/s12883-024-03647-7)
Supplement: Supplementary file 1 — Supplementary Material 1 [file 12883_2024_3647_MOESM1_ESM.docx]

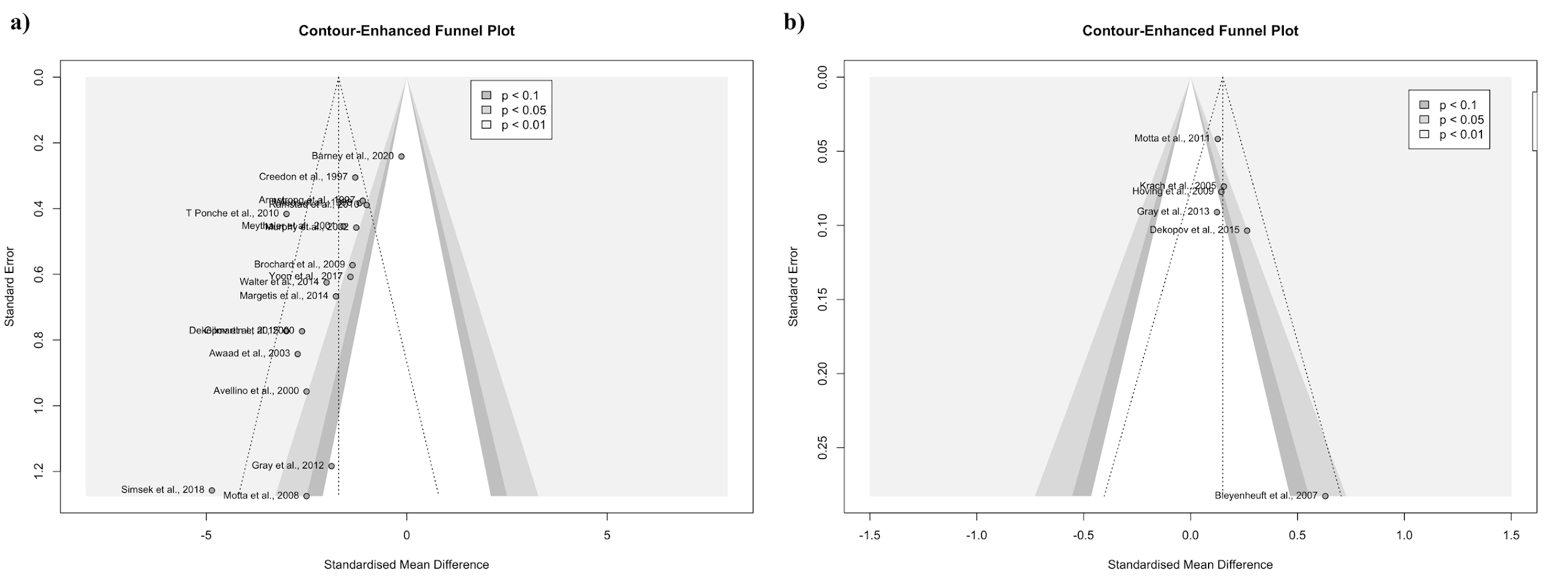


**Supplementary Figure 1.** Funnel plots; (a) for severity of spasticity and (b) for motor function

**Supplementary Table 1.** Search strategy using the PICO model

**Supplementary Table 2.** Every complication and adverse event reported in the included studies

| Author | Number of complications and adverse events | Total number of participants* | Reference |
| --- | --- | --- | --- |
| Brochard et al. | Cutaneous hematoma: 1, Catheter disconnection: 1 | 7 participants | ^1^ |
| Simsek et al. | Varying swelling: 1 | 22 participants | ^2^ |
| Ramstad et al. | Intolerable agitation: 1, Infections: 2*Leading to the discontinuation of ITB treatment in all three cases | 38 participants | ^3^ |
| Meythaler et al. | Post-ITB scoliosis surgery: 2, Constipation: 2, Headache and nausea: 2, New-onset seizures: 2 | 13 participants | ^4^ |
| Bleyenheuft et al. | Chemical meningitis: 1, Cerebrospinal fluid leaks: 2 | 7 participants | ^5^ |
| Awaad et al. | Nausea: 4, Constipation: 6, Increased seizure frequency: 2, New-onset seizures: 2, Increased oral secretions: 2, Sleepiness: 2, Urinary retention: 1, Meningitis: 2, Seroma: 7, Pump rotation: 3, Infection: 3, Catheter dislocation: 3 | 29 participants | ^6^ |
| Gray et al. | Pump or catheter complications: 4, Constipation: 11, Urinary retention: 2, Lethargy: 2, New-onset seizures: 2, Jumpy legs: 1, Hypothermia: 1, Increased oral secretions: 1, Reflux: 1, Pressure sore: 2, Nausea: 1, Swallowing problem: 2 | 37 participants | ^7^ |
| Motta et al. | Catheter complications: 3, Infection: 1, Cerebrospinal fluid leaks: 1 | 37 participants | ^8^ |
| Gilmartin et al. | Seroma: 7, Infection: 5, Cerebrospinal fluid leaks: 3, Catheter complications: 8 | 44 participants | ^9^ |
| Avellino et al. | Catheter complications: 13, Infection: 5, Sleepiness: 1, Psychiatric side effects: 1, Increased seizure frequency: 1, Cerebrospinal fluid leaks: 1, Pump complications: 1 | 62 participants | ^10^ |
| Margetis et al. | Pump or catheter complications: 3 | 15 participants | ^11^ |
| Tassëel Ponche et al. | Psychiatric side effects: 1, Infection: 1, Constipation: 1, Cerebrospinal fluid leaks: 2, Headaches, nausea, vomiting: 3, Pressure sore: 1, Urinary incontinence: 2, Dysuria: 1, Swallowing problem: 2, Pruritus: 1, Leg edema: 1, Catheter complications: 1 | 25 participants | ^12^ |
| Murphy et al. | Cerebrospinal fluid leaks: 4, Vomiting: 3, Headaches: 3, Wound infection/dehiscence: 9, Meningitis: 2, Catheter complications: 6 | 25 participants | ^13^ |
| Walter et al. | Infection: 3, Seroma: 1, Skin problems: 2, Meningitis: 1, Pump complications: 2, Overdose: 1 | 15 participants | ^14^ |
| Armstrong et al. | Hypotension: 2, Bradycardia: 2, Sedation: 1, Respiratory depression:1, Apnea: 1, Progression of scoliosis: 2, Increased seizure frequency: 3, Meningitis: 2, Infection: 3, Pump or catheter complications: 10 | 19 participants | ^15^ |
| Yoon et al. | Headache: 1, Drowsiness: 2, Decreased balance: 2, Cerebrospinal fluid leaks: 1, Catheter complications: 2, Infection: 1, Withdrawal symptoms: 1, Wound dehiscence: 1 | 37 participants | ^16^ |
| Dekopov et al. | Seroma: 4 | 15 participants | ^17^ |
| Hoving et al. | Wound leakage: 1, Cerebrospinal fluid leaks: 2, Varying swelling: 10, Pruritus: 4, Cystitis: 1, Pump or catheter complications: 6 | 17 participants | ^18^ |
| Wiens et al. | Pump or catheter complications: 6, Fluid leaks or seromas: 5, Flaccidity, irritability, sleeplessness, and hallucinations: 5, Increased seizures frequency: 1 | 17 participants | ^19^ |
| Motta et al. | Catheter complications: 3 | 20 participants | ^20^ |

# * The total number of participants differs from what is stated in the meta-analysis since some patients did not have cerebral palsy and were thus excluded from the meta-analysis, but are included here for the purpose of evaluating complication prevalence.

1. Brochard, S., Lempereur, M., Filipetti, P. & Rémy-Néris, O. Changes in gait following continuous intrathecal baclofen infusion in ambulant children and young adults with cerebral palsy. *Dev Neurorehabil* 12, 397–405 (2009).

2. Simsek, H., Zorlu, E., Bakal, O., Akarsu, S. & Güney Senol, M. Continuous intrathecal baclofen delivery in severely disabling spasticity. *Vojnosanit Pregl* 75, 1076–1082 (2018).

3. Ramstad, K., Jahnsen, R., Lofterod, B. & Skjeldal, O. H. Continuous intrathecal baclofen therapy in children with cerebral palsy - when does improvement emerge? *Acta Paediatr* 99, 1661–1665 (2010).

4. Meythaler, J. M., Guin-Renfroe, S., Law, C., Grabb, P. & Hadley, M. N. Continuously infused intrathecal baclofen over 12 months for spastic hypertonia in adolescents and adults with cerebral palsy. *Arch Phys Med Rehabil* 82, 155–161 (2001).

5. Bleyenheuft, C., Filipetti, P., Caldas, C. & Lejeune, T. Experience with external pump trial prior to implantation for intrathecal baclofen in ambulatory patients with spastic cerebral palsy. *Neurophysiol Clin* 37, 23–28 (2007).

6. Awaad, Y. *et al.* Functional assessment following intrathecal baclofen therapy in children with spastic cerebral palsy. *J Child Neurol* 18, 26–34 (2003).

7. Gray, N., Morton, R. E., Brimlow, K., Keetley, R. & Vloeberghs, M. Goals and outcomes for non ambulant children receiving continuous infusion of intrathecal baclofen. *Eur J Paediatr Neurol* 16, 443–448 (2012).

8. Motta, F., Antonello, C. E. & Stignani, C. Intrathecal baclofen and motor function in cerebral palsy. *Dev Med Child Neurol* 53, 443–448 (2011).

9. Gilmartin, R. *et al.* Intrathecal baclofen for management of spastic cerebral palsy: multicenter trial. *J Child Neurol* 15, 71–77 (2000).

10. Avellino, A. M. & Loeser, J. D. Intrathecal baclofen for the treatment of intractable spasticity of spine or brain etiology. *Neuromodulation* 3, 75–81 (2000).

11. Margetis, K. *et al.* Intrathecal baclofen improves psychiatric symptoms in spasticity patients. *J Clin Psychopharmacol* 34, 374–379 (2014).

12. Tassëel Ponche, S. *et al.* Intrathecal baclofen in cerebral palsy. A retrospective study of 25 wheelchair-assisted adults. *Ann Phys Rehabil Med* 53, 483–498 (2010).

13. Murphy, N. A., Irwin, M. C. N. & Hoff, C. Intrathecal baclofen therapy in children with cerebral palsy: efficacy and complications. *Arch Phys Med Rehabil* 83, 1721–1725 (2002).

14. Walter, M., Altermatt, S., Furrer, C. & Meyer-Heim, A. Intrathecal baclofen therapy in children with severe spasticity: Outcome and complications. *Dev Neurorehabil* 17, 368–374 (2014).

15. Armstrong, R. W. *et al.* Intrathecally administered baclofen for treatment of children with spasticity of cerebral origin. *J Neurosurg* 87, 409–414 (1997).

16. Yoon, Y. K. *et al.* Outcomes of intrathecal baclofen therapy in patients with cerebral palsy and acquired brain injury. *Medicine* 96, (2017).

17. Dekopov, A. V., Shabalov, V. A., Tomskiy, A. A., Gaevyi, I. O. & Salova, E. M. Preliminary results of chronic intrathecal therapy in treatment of spastic syndromes of various etiologies. *Zh Vopr Neirokhir Im N N Burdenko* 79, 27–33 (2015).

18. Hoving, M. A. *et al.* Safety and one-year efficacy of intrathecal baclofen therapy in children with intractable spastic cerebral palsy. *Eur J Paediatr Neurol* 13, 247–256 (2009).

19. Wiens, H. D. Spasticity in children with cerebral palsy: a retrospective review of the effects of intrathecal baclofen. *Issues Compr Pediatr Nurs* 21, 49–61 (1998).

20. Motta, F., Stignani, C. & Antonello, C. E. Upper limb function after intrathecal baclofen treatment in children with cerebral palsy. *J Pediatr Orthop* 28, 91–96 (2008).
